# POs‐Ca Drives Osteogenic Differentiation of Human Dental Pulp Stem Cells Via AMPK‐Dependent Autophagy Activation and Reciprocal Calcium‐Autophagy Crosstalk

**DOI:** 10.1155/sci/6150093

**Published:** 2025-12-29

**Authors:** Jiayuan Zhang, Yunqing Liu, Shuhei Hoshika, Chiharu Kawamoto, Hidehiko Sano, Atsushi Tomokiyo, Jie Gao, Sujit Nair

**Affiliations:** ^1^ Department of Restorative Dentistry, Graduate School of Dental Medicine, Hokkaido University, Sapporo, Japan, hokudai.ac.jp; ^2^ Department of Stomatology, The Fourth Affiliated Hospital of Harbin Medical University, Harbin, China, hrbmu.edu.cn; ^3^ Department of Restorative Dentistry, Faculty of Dental Medicine, Hokkaido University, Sapporo, Japan, hokudai.ac.jp; ^4^ Department of Peripheral Vascular Diseases, First Affiliated Hospital, Heilongjiang University of Traditional Chinese Medicine, Harbin, China, hljucm.edu.cn

**Keywords:** AMPK signaling pathway, autophagy, calcium signaling, hDPSCs, osteogenic differentiation, POs-Ca

## Abstract

Human dental pulp stem cells (hDPSCs) hold significant promise for bone regeneration, yet efficient osteogenic induction remains challenging. Phosphorylated oligosaccharides of calcium (POs‐Ca), a novel calcium salt derived from potato starch, has recently attracted attention for its remineralization capabilities and potential to promote stem cell differentiation. Here, we investigated the impact of POs‐Ca on the osteogenic differentiation of hDPSCs and its underlying mechanism. Isolated hDPSCs were characterized via flow cytometry based on mesenchymal surface markers. Biocompatibility and osteogenic differentiation were assessed via Cell Counting Kit‐8 (CCK‐8) assay, alkaline phosphatase (ALP) activity, Alizarin Red S staining, and protein levels of osteogenic (Collagen I, DSPP, DMP1, and RUNX2). Intracellular Ca^2+^ flux was monitored using Fluo‐4 AM, while AMP‐activated protein kinase (AMPK) signaling and autophagic flux were analyzed by western blot (p‐AMPK, p‐ULK1, and light chain 3 (LC3)‐II), TEM, and LC3‐GFP imaging. Mechanistic studies employed verapamil (Ca^2+^ channel blocker), Compound C (CC;AMPK inhibitor), and chloroquine (CQ;autophagy inhibitor). POs‐Ca (5 mg/mL) exhibited excellent biocompatibility and significantly promoted osteogenic differentiation, as evidenced by a 3.22‐fold increase in ALP activity and markedly enhanced mineralized nodule formation as shown by Alizarin Red S staining. Mechanistic studies revealed that POs‐Ca triggers rapid intracellular Ca^2+^ influx, activating the AMPK pathway and inducing autophagic flux. Pharmacological inhibition established the essential causality of this cascade: verapamil abolished osteogenic enhancement, while CC and CQ suppressed ALP activity, mineralization, and osteogenic marker expression. Notably, CQ reciprocally attenuated POs‐Ca‐induced Ca^2+^ influx, revealing novel bidirectional Ca^2+^‐autophagy crosstalk. In conclusion, POs‐Ca might promote hDPSCs osteogenesis via a calcium influx‐driven AMPK‐autophagy axis, providing a foundation for novel biomaterials that exploit physiological calcium signaling. These findings offer immediate translational potential for developing minimally invasive, cost‐effective strategies in dental pulp regeneration and bone defect repair.

## 1. Introduction

Human dental pulp stem cells (hDPSCs), a well‐characterized population of mesenchymal stem cells (MSCs) residing within the dental pulp tissue [1, 2], possess remarkable self‐renewal and the ability to differentiate into multiple lineages, including osteogenic, adipogenic, and neurogenic lineages [3]. Their relative accessibility via minimally invasive procedures (e.g., extraction of third molars) and intrinsic regenerative capabilities position hDPSCs as a highly promising autologous cell source for applications in craniofacial and orthopedic bone regeneration [4–6]. However, a critical barrier hindering their clinical translation is the need for efficient and controllable induction of osteogenic differentiation. Current strategies often rely on supraphysiological doses of exogenous growth factors (e.g., BMP‐2) within complex scaffolds [7–9]. While demonstrably effective, this approach faces significant limitations, including high cost, potential ectopic bone formation, immune reactions, and short biological half‐lives necessitating repeated administration [10]. Consequently, there is a pressing need to develop novel, cost‐effective, and biocompatible bioactive materials capable of physiologically triggering and sustaining the intrinsic osteogenic program of hDPSCs.

Calcium‐based biomaterials have emerged as promising candidates due to the dual role of calcium ions (Ca^2+^) as both mineralization components and signaling molecules. Extracellular Ca^2+^ can trigger intracellular signaling by activating receptors such as the calcium‐sensing receptor (CaSR), promoting osteogenic differentiation [11–13]. Nevertheless, conventional calcium phosphates (e.g., hydroxyapatite and *β*‐tricalcium phosphate[TCP]) often suffer from slow dissolution kinetics and predominantly passive osteoconduction, limiting their bioactivity in actively directing stem cell fate [14]. This underscores the need for novel calcium delivery platforms with enhanced solubility and intrinsic signaling capacity.

Phosphorylated oligosaccharides of calcium (POs‐Ca), a novel water‐soluble calcium salt derived from potato starch—is a widely available, low‐cost, and renewable agricultural feedstock. POs‐Ca uniquely combines the delivery of bioavailable Ca^2+^ ions with acidic oligosaccharides [15, 16]. Unlike poorly soluble calcium phosphates, POs‐Ca contains ~5% (w/w) calcium and exhibits excellent aqueous solubility. It also demonstrates remarkable physicochemical stability across a wide range of temperatures and pH values commonly encountered in food and oral formulations, supporting its incorporation into biofunctional products. Its prior safe application in consumer goods, such as sugar‐free chewing gum, soft candy, and fruit juices, reinforces its biocompatibility and regulatory accessibility, providing a practical foundation for therapeutic development. Notably, POs‐Ca has shown well‐documented biological activities [17–19], particularly in dental applications, where it enhances enamel remineralization and synergistically improves fluoride efficacy under physiological pH conditions. Its dual ability to promote mineral deposition and stabilize pH [20–24], along with its high bioavailability, positions it as a promising adjunct in fluoride‐based caries prevention and enamel repair strategies [22]. Building on this foundation, we hypothesize that POs‐Ca may serve as a potent bioactive agent in stem cell‐based therapies by releasing bioavailable Ca^2+^ ions to directly modulate intracellular signaling pathways in hDPSCs, thereby stimulating their osteogenic differentiation.

Given that intracellular Ca^2+^ not only contributes to mineralization but also serves as a second messenger that interfaces with key metabolic regulators, it is essential to explore its downstream molecular targets in the context of stem cell differentiation. One such key effector is AMP‐activated protein kinase (AMPK), a master heterotrimeric serine/threonine kinase regulating cellular energy homeostasis [25]. AMPK can be activated by increases in cytosolic Ca^2+^ via upstream kinases, notably Ca^2+^/calmodulin‐dependent protein kinase kinase *β* (CaMKK*β*) [26, 27]. Beyond its classical roles in energy sensing, activated AMPK orchestrates diverse cellular responses, including the induction of autophagy—a critical lysosomal degradation pathway essential for cellular remodeling, organelle quality control, and stem cell fate determination [28]. Emerging evidence implicates both AMPK activation and functional autophagy as positive regulators of osteogenic differentiation in MSCs [29–32]. However, the specific interplay between Ca^2+^ influx, AMPK activation, autophagy induction, and osteogenesis in hDPSCs, particularly in response to bioactive calcium‐delivering agents like POs‐Ca, remains largely unexplored and represents a significant knowledge gap.

This study demonstrates that POs‐Ca, a bioavailable calcium‐delivering agent, potently enhances osteogenic differentiation of hDPSCs by activating a novel AMPK‐dependent autophagy axis triggered by calcium influx, providing a mechanistic foundation for advanced biomaterial strategies in bone and dental regeneration.

## 2. Materials and Methods

### 2.1. Cell Culture and Characterizations of hDPSCs

hDPSCs were isolated from sound third molars of healthy donors (age 16–25, *n* = 10) with informed consent and institutional approval (Hokkaido University Ethics Committee, protocol #2018−09). Teeth were rinsed in sterile phosphate‐buffered saline (PBS) containing 1% penicillin/streptomycin (Sigma–Aldrich, MO, USA), split longitudinally with a sterile chisel, and dental pulp tissues were aseptically isolated under a laminar‐flow hood. Pulp samples were minced into ~1 mm^3^ fragments and digested in 3 mg/mL collagenase type I (Gibco, Grand Island, NY, USA) at 37°C with gentle agitation. Following digestion, the cell suspension was passed through a 70 *μ* m cell strainer, centrifuged at 300 × *g* for 5 min, and resuspended in growth medium. Cells were cultured in DMEM/F‐12 medium (Thermo Fisher Scientific, MA, USA) supplemented with 20% fetal bovine serum (FBS, Thermo Fisher Scientific), and1% penicillin/streptomycin (Sigma–Aldrich, MO, USA) and maintained at 37°C in a humidified atmosphere of 5% CO_2_. The culture medium was changed every 2–3 days. Through successive subculture, cells were expanded for further experiments (passage 3–5).

For characterization, 1 × 10^5^ cells were harvested using 0.25% trypsin‐EDTA (Hyclone, UT, USA) and suspended in 100 mL of PBS containing 1% FBS. To confirm their phenotype, cells were labeled with antibodies against MSC markers CD44, CD29, CD90, and CD105, and hematopoietic markers CD34 as well as CD45 (BioLegend, San Diego, CA, USA), following standard protocols for flow cytometric analysis [33]. The following antibodies were used: CD44 (APC, Clone IM7, 1:100), CD29 (PE, Clone MAR4, 1:100), CD90 (APC, Clone 5E10, 1:100), CD105 (PE, Clone MJ7/18, 1:100), CD34 (PE, Clone 563, 1:100), and CD45 (FITC, Clone HI30, 1:100). The expression of these markers was then assessed by a FACSCalibur flow cytometer (BD Biosciences, CA, USA). The data were analyzed using FlowJo software (FlowJo LLC, Ashland, OR, USA).

### 2.2. POs‐Ca Preparation and Cell Viability Assay

POs‐Ca was kindly provided by Glico Nutrition Co., Ltd (Osaka, Japan). For cell treatment, POs‐Ca powder was suspended in complete culture medium and sonicated for 15 min to achieve uniform dispersion. The suspension was freshly prepared and sterilized using a 0.22‐*μ* m filter prior to each experiment. The control group was treated with PBS under the same conditions. Cell Counting Kit‐8 (CCK‐8; Dojindo, Japan) was performed to assess the cell viability of hDPSCs in the presence of POs‐Ca. For this analysis, 1 × 10^4^ cells/well were seeded into 96‐well plates and cultured in DMEM/F‐12 supplemented with 10% FBS, and 1% penicillin/streptomycin for 24 h until reaching confluency. The cells were then exposed to varying concentrations of POs‐Ca (0, 1, 2, 4, and 8 mg/mL) for 1, 4, 7, and 14 days. The culture medium was refreshed every 2–3 days throughout the treatment period. After treatment, the medium was removed, and the cells were washed with PBS for three times. Then, 100 *μ* L of culture medium containing 10 *μ* L of CCK‐8 solution was added to each well. The plates were incubated at 37°C protected from light for 2 h according to the manufacturer’s instructions. Absorbance at 450 nm was measured using a microplate reader (Bio‐Rad, CA, USA). This experiment was repeated at least three times. The results were normalized to the untreated control group and expressed as a percentage of relative cell viability.

### 2.3. Cell Apoptosis Assay

To access the effect of POs‐Ca on the apoptosis of hDPSCs, cells were seeded at 1 × 10^5^ cells per well in 6‐well plates and grown in basal growth medium until confluence. Then, the cells were then exposed to POs‐Ca for 1, 4, and 7 days. Following the exposure, cells were harvested with 0.25% trypsin/EDTA (Invitrogen Life Technologies) and resuspended in binding buffer (BD Biosciences, Franklin Lakes, NJ, USA). Apoptotic cells were evaluated using an annexin V‐FITC/propidium iodide (PI) double staining assay (BD, Bergen, NJ, USA) according to the manufacturer’s instructions. Cell samples were then analyzed by flow cytometry (Beckman Coulter, Brea, CA, USA). The proportions of annexin V‐positive cells were recorded as apoptotic rates. Data were analyzed using FlowJo software (FlowJo LLC, Ashland, OR, USA).

### 2.4. Osteogenic and Adipogenic Differentiation Assays

For osteogenic induction, hDPSCs were seeded in 48‐well plates and cultured in standard osteogenic induction medium supplemented with 10 mM *β*‐glycerophosphate, 50 µg/mL ascorbic acid, and 10 nM dexamethasone, with or without the indicated concentration of POs‐Ca. To inhibit AMPK activity, a selective AMPK inhibitor Compound C (CC, 5 µM, Sigma–Aldrich) was added to the medium to pretreat the cells for 1 h before the addition of POs‐Ca. Medium was changed every 3 days, and cells were cultured at 37°C in a 5% CO_2_ incubator.

After 7 days, alkaline phosphatase (ALP) activity was measured using a commercial kit (Beyotime Biotechnology, Zhejiang, China) according to the manufacturer’s instructions [34]. The total protein content was quantified simultaneously by a BCA protein assay kit (Thermo Fisher Scientific), and ALP activity was normalized to protein concentration. Absorbance at 405 nm was measured using a microplate reader.

To assess the mineralization, hDPSCs were cultured under the same osteogenic conditions for 21 days. Cells were washed once with PBS and fixed with 4% paraformaldehyde (Sigma–Aldrich) for 15 min at room temperature. Mineralization was assessed by staining with Alizarin Red S staining solution (2%, pH 4.2) following the manufacturer’s instructions (Beyotime Biotechnology, China). Excess dye was removed by gentle washing with deionized water. Calcium‐rich mineralized nodules were visualized by light microscopy (Olympus, IX71, Japan).

For adipogenic differentiation, cells were cultured in adipogenic induction medium (DMEM/F‐12 supplemented with 10% FBS, 1% penicillin/streptomycin, 10 µg/mL insulin, 1 µM dexamethasone, and 0.5 mM isobutylmethylxanthine) for 14 days. Lipid accumulation was visualized using Oil Red O staining, as described previously [35].

### 2.5. Calcium Influx Analysis

To delineate the regulatory role of intracellular calcium flux in hDPSCs osteogenesis, we employed verapamil (Sigma–Aldrich)—a pharmacological blocker of voltage‐gated calcium channels (VGCCs). Cells were seeded in 6‐well plates (1 × 10^5^ cells/well) and cultured in basal growth medium until reaching confluence. Confluent cultures were pre‐treated with 10 *μ* M verapamil for 1 h prior to osteogenic induction to ensure effective channel blockade. Following this pharmacological intervention, cells were maintained under osteogenic conditions and concurrently exposed to POs‐Ca treatment for 24 h. Intracellular calcium levels were measured using the Ca^2+^‐sensitive fluorescent dye Fluo‐4 AM (Invitrogen, CA, USA) according to the manufacturer’s protocol. Briefly, cells were loaded with Fluo‐4 AM (2 µM) for 30 min at 37°C, followed by an additional 30 min incubation at room temperature. After three washes with PBS, cells nuclei were counterstained with 4’,6‐diamidino‐2‐phenylindole (DAPI;Sigma–Aldrich) for 10 min at room temperature. All samples were imaged using fluorescence microscopy (Nikon, Tokyo, Japan).

### 2.6. Transmission Electron Microscopy (TEM)

After cell treatment, cells were washed three times with PBS, then fixed with 2.5% glutaraldehyde in 0.1 M phosphate buffer (pH 7.4) for 2 h at 4°C, postfixed with 1% osmium tetroxide for 1 h, dehydrated through an ethanol gradient (50%, 70%, 80%, 90%, 95%, and 100%), dried with hexamethyldisilazane (HMDS), and embedded in Epon 812. Ultrathin sections (70 nm) were cut using an ultramicrotome (Leica EM UC7, Wetzlar, Germany), stained with 2% uranyl acetate for 15 min and lead citrate for 10 min, and examined using a TEM (JEOL JEM‐1400 Plus, Japan) operating at 80 kV. For quantification of autophagosomes, 20 cells per condition were randomly selected, and the number of autophagosomes per cell was counted.

### 2.7. Detection of Autophagic Flux by Light Chain 3 (LC3)‐GFP Analysis

To elucidate the functional role of autophagy in osteogenic differentiation of hDPSCs, pharmacological inhibition was performed using chloroquine (CQ, 5 *μ* M; Sigma–Aldrich), a lysosomal acidification inhibitor that blocks autophagosomelysosome fusion. Autophagic activity was quantitatively assessed using the FlowCellect LC3‐GFP Reporter Autophagy Assay Kit (EMD Millipore, Bedford, MA, USA) according to established protocols. hDPSCs were plated in 6‐well plates at a density of 1 × 10^5^ cells/well and cultured to 70%–80% confluence. Cells were transiently transfected with the FlowCellect LC3‐GFP Reporter plasmid using Lipofectamine 3000 transfection reagent (Invitrogen) under optimized conditions as per manufacturer specifications. This construct enables real‐time monitoring of autophagosome formation through GFP‐tagged microtubule‐associated protein 1A/1B‐LC3.

Following a 24‐h post‐transfection recovery period, osteogenic induction was initiated by supplementing with POs‐Ca, with parallel treatment groups exposed to CQ (5 *μ* M) or vehicle control. After 24 h of combined osteogenic/autophagy‐modulatory treatment, cells were harvested via trypsinization and washed twice with PBS and fixed in 4% paraformaldehyde (Sigma–Aldrich) for 10 min at room temperature. Fixed cells underwent three PBS washes to eliminate residual fixative prior to nuclear counterstaining with DAPI (0.1 *μ* g/mL, Sigma–Aldrich) for 10 min at room temperature. Specimens were then mounted using Fluoromount‐G and visualized under a fluorescence microscope (Nikon) with an excitation wavelength of 488 nm and an emission wavelength of 509 nm for GFP detection. Autophagy levels were assessed by manually counting the number of LC3‐positive puncta per cell.

### 2.8. Western Blot Analysis

For protein expression analysis, total proteins were extracted using RIPA lysis buffer (Beyotime Biotechnology, China) supplemented with protease inhibitors (Roche Applied Sciences, Basel, Switzerland). Protein concentrations were determined using the BCA protein assay kit (Thermo Fisher Scientific). Further, equal amounts of proteins (30 µg) were separated by SDS–polyacrylamide gel electrophoresis (SDS‐PAGE, 10%) and transferred to polyvinylidene fluoride (PVDF) membranes (EMD Millipore, Bedford, MA, USA) by electrophoresis. Following blocking in 5% skimmed milk (dissolved in TBST, TBS with 0.1% Tween‐20) for 1 h at room temperature, the membranes were incubated with primary antibodies at 4°C overnight. The primary antibodies detected in immunoblotting were listed as follows: anti‐LC3B antibody (Sigma–Aldrich, St. Louis, MO, USA), anti‐phospho–AMPK antibody (Abcam, Cambridge, UK), anti‐DSPP (Santa Cruz Biotechnology, Dallas, TX, USA), anti‐DMP1 antibody (Abcam, Cambridge, UK), anti‐phospho–ULK1 antibody (Proteintech Group, Wuhan, China), anti‐Collagen I antibody (Abcam, Cambridge, UK), and anti‐RUNX2 antibody (MBL, Woburn, MA, USA). anti‐GAPDH antibody (Rayantibody, Beijing, China) was used as an internal control. After being washed with 1 × TBST three times for 10 min, the membranes were then incubated with horseradish peroxidase‐conjugated secondary antibodies (Cat No.: CW0103S, CWBiotech, China) for incubation 1.5 h at room temperature with gentle shaking. The membranes were washed three times with 1 × TBST for 10 min. The blots were then visualized using an ECL detection system (Bio‐Rad).

### 2.9. Statistical Analysis

All experiments were performed in triplicate, with at least three independent biological replicates. Data were presented as mean ± standard deviation. Statistical analysis was performed using GraphPad Prism 8.0 software. One‐way ANOVA followed by Tukey’s post hoc test was used for multiple comparisons. For experiments comparing only two groups, Student’s *t*‐test was used. *p* < 0.05 was considered statistically significant.

## 3. Results

### 3.1. Characterization of Isolated hDPSCs

Flow cytometry analysis revealed that the hDPSCs originating from sustained explant culture exhibited a characteristic MSC phenotype, with high expression of CD44 (99.8%), CD29 (98.6%), CD90 (98.5%), and CD105 (97.3%). Minimal expression was observed for hematopoietic markers CD34 (2.56%) and CD45 (2.63%) (Figure 1A). The osteogenic differentiation potential of hDPSCs was confirmed by Alizarin Red S staining, which demonstrated significant calcium deposition following osteogenic induction (Figure 1B). Additionally, Oil Red O staining revealed the formation of lipid droplets after adipogenic induction, confirming the adipogenic differentiation potential of hDPSCs (Figure 1C). Collectively, these results confirm the successful isolation of MSC from dental pulp with multilineage differentiation potential.

Figure 1Characterization and biocompatibility assessment of hDPSCs. (A) Flow cytometric analysis of surface antigen expression in isolated hDPSCs. Cells were labeled with antibodies against mesenchymal stem cell markers (CD29, CD44, CD90, and CD105) and hematopoietic markers (CD34 and CD45); percentages of positive cells are indicated. (B) Osteogenic differentiation potential of hDPSCs. Cells were cultured in control or osteogenic induction medium for 21 days and stained with Alizarin Red S to detect calcium deposition. Scale bar = 100 μ m. (C) Adipogenic differentiation potential of hDPSCs. Cells were cultured in control or adipogenic induction medium for 14 days and stained with Oil Red O to visualize lipid droplets. Scale bar = 100 μ m.

**Figure:**
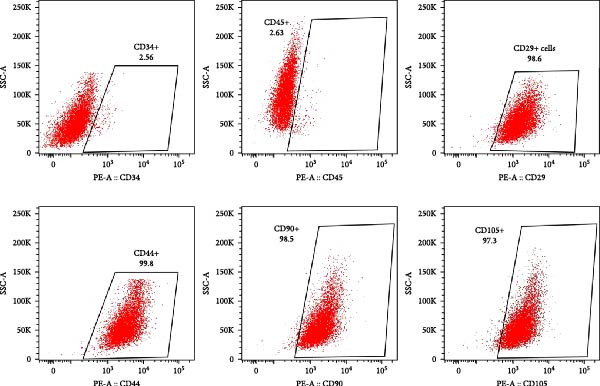
(A)

**Figure:**
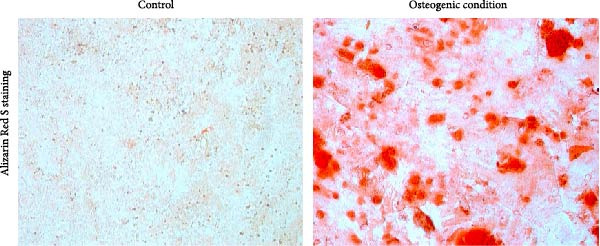
(B)

**Figure:**
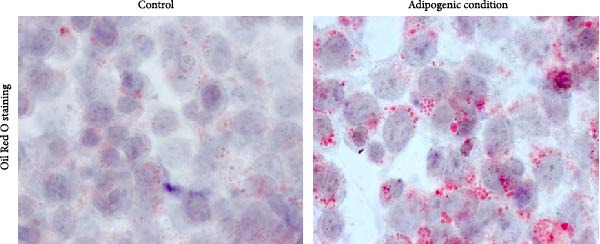
(C)

### 3.2. Biocompatibility of POs‐Ca: Effects on hDPSC Viability and Apoptosis

Following phenotypic confirmation of hDPSCs, we assessed the biocompatibility of POs‐Ca. To assess potential induction of apoptosis, hDPSCs treated with 5 mg/mL POs‐Ca were analyzed by flow cytometry using annexin V‐FITC/PI double staining. Dot plots defined viable cells as annexin V^-^/PI^-^ (lower left), early apoptotic cells as annexin V^+^/PI^-^ (lower right), necrotic cells as annexin V^-^/PI^+^ (upper left), and late apoptotic cells as annexin V^+^/PI^+^ (upper right) (Figure 2A). Treatment with POs‐Ca induced no significant change in the combined percentage of early and late apoptotic cells over 7 days. Quantitative analysis from three independent experiments confirmed that the total apoptotic cell fraction (early + late apoptosis) remained comparable between the POs‐Ca‐treated group and control group at days 1, 4, and 7 (*p* > 0.05, Figure 2A).

Figure 2Effects of POs‐Ca on hDPSCs viability and apoptosis. (A) Representative flow cytometry dot plots showing annexin V‐FITC/PI staining of control and POs‐Ca‐treated (5 mg/mL) hDPSCs at days 1, 4, and 7. Cells were stained with annexin V‐FITC and propidium iodide (PI) to discriminate early apoptotic (annexin V^+^/PI^-^) and late apoptotic/necrotic (annexin V^+^/PI^+^) populations. Quantification of total apoptosis (early + late apoptotic cells) revealed no significant differences between control and POs‐Ca‐treated groups at any time point. Data were presented as mean ± SD (*n* = 3). Statistical comparison was performed using two‐way ANOVA with Sidak’s multiple comparisons test (*p* > 0.05). (B) Dose‐dependent cytotoxicity of POs‐Ca on hDPSCs evaluated by CCK‐8 assay after 24 h of treatment. The half‐maximal inhibitory concentration (IC_50_) was determined, and 5 mg/mL was identified as a noncytotoxic concentration used in subsequent experiments. (C) Long‐term viability of hDPSCs following exposure to 5 mg/mL POs‐Ca, measured by CCK‐8 assay on days 1, 4, 7, and 14. Data were presented as mean ± SD from three independent experiments.

**Figure:**
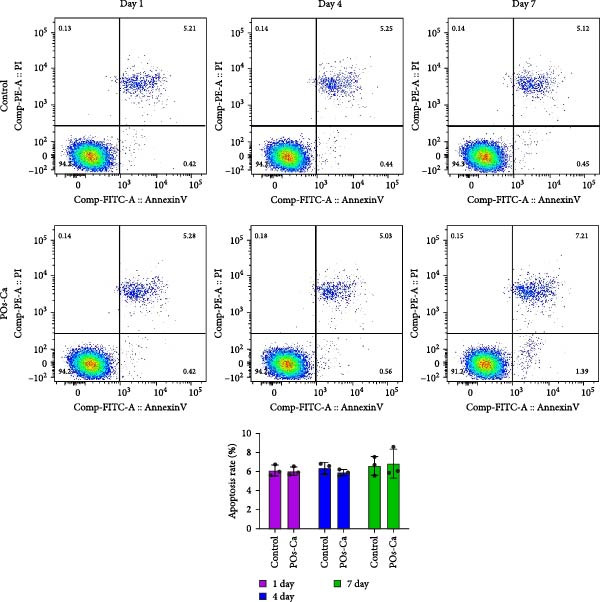
(A)

**Figure:**
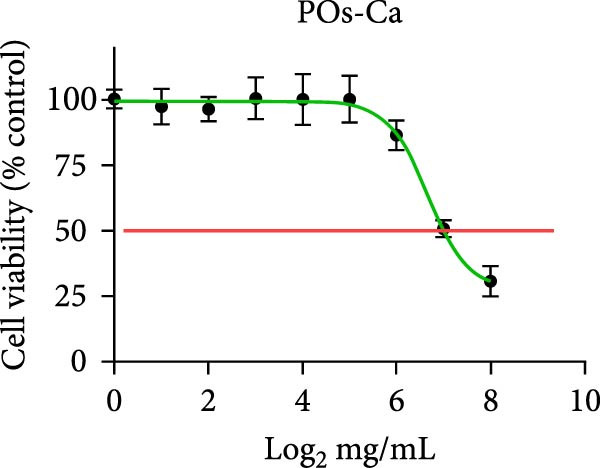
(B)

**Figure:**
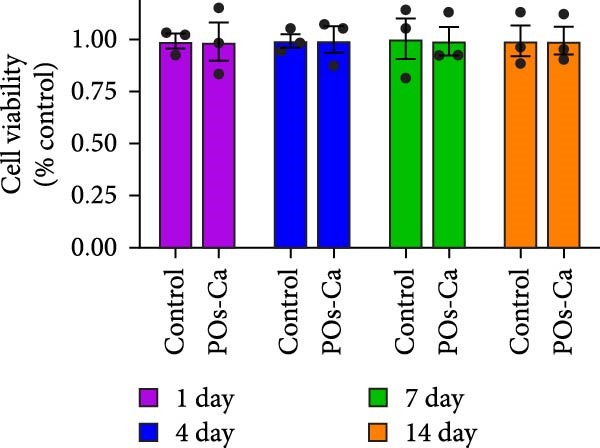
(C)

In the dose–response assay, treatment with POs‐Ca at concentrations up to 5 mg/mL for 24 h maintained high cell viability (99.28 ± 6.34% of control), with no significant difference versus untreated cells (*p* > 0.05, Figure 2B). Cytotoxicity became evident at concentrations exceeding 5 mg/mL, yielding a calculated IC_50_ of 6.68 ± 0.12 mg/mL (four‐parameter logistic regression, *R* ^2^ = 0.9495, Figure 1B). Based on these findings, 5 mg/mL was chosen as the maximal nontoxic concentration for all subsequent experiments. Long‐term viability assays confirmed that continuous exposure to 5 mg/mL POs‐Ca over 14 days did not compromise hDPSCs survival (*p* > 0.05, Figure 2C).

These results collectively demonstrate that POs‐Ca, at the concentration of 5 mg/mL, exhibits excellent biocompatibility with hDPSCs, inducing neither cytotoxicity nor apoptosis.

### 3.3. POs‐Ca Induces Intracellular Calcium Influx and Promotes Osteogenic Differentiation of hDPSCs

We next investigated the biological activity of POs‐Ca on hDPSCs, focusing initially on intracellular calcium dynamics, a key regulator of cellular metabolism and differentiation. Fluo‐4 AM fluorescence imaging revealed that treatment with 5 mg/mL POs‐Ca induced a rapid and marked increase in intracellular calcium levels compared to untreated controls (Figure 3A), indicating enhanced calcium influx upon POs‐Ca exposure. However, this calcium influx was significantly attenuated by pretreatment with the calcium channel blocker verapamil, confirming the role of transmembrane calcium entry in the POs‐Ca‐mediated response.

Figure 3Calcium influx mediates POs‐Ca‐induced osteogenic differentiation in hDPSCs. (A) Representative fluorescence images of hDPSCs stained with Fluo‐4 AM (green) for intracellular calcium signaling and DAPI (blue) for nuclei after 24‐h treatment. POs‐Ca (5 mg/mL) markedly increased calcium influx compared to control, while cotreatment with verapamil, a calcium channel blocker, attenuated this effect. Scale bar = 50 μm. (B) Quantification of alkaline phosphatase (ALP) activity in hDPSCs after 7 days of treatment. POs‐Ca significantly increased ALP activity, which was partially reversed by verapamil, indicating calcium influx involvement in early osteogenic induction. Data were presented as mean ± SD from three independent experiments.  ^∗^ *p* < 0.05 compared to control; #*p* < 0.05 compared to POs‐Ca group. (C) Representative images of Alizarin Red S staining for mineralized matrix after 21 days. POs‐Ca treatment enhanced mineral deposition, whereas verapamil cotreatment reduced the mineralization. Scale bar = 100 μm. (D) Western blot analysis of osteogenic markers (collagen I, DSPP, DMP1, and RUNX2) in POs‐Ca‐treated, and POs‐Ca + verapamil‐treated hDPSCs after 7 days. POs‐Ca upregulated the expression of all markers, while verapamil inhibited these effects. Data were presented as mean ± SD from three independent experiments.  ^∗^ *p* < 0.05 compared to control;  ^∗∗^ *p* < 0.01 compared to POs‐Ca group.

**Figure:**
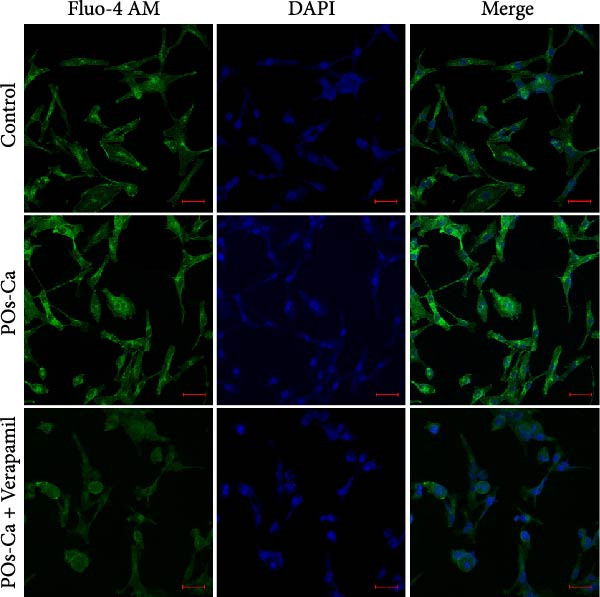
(A)

**Figure:**
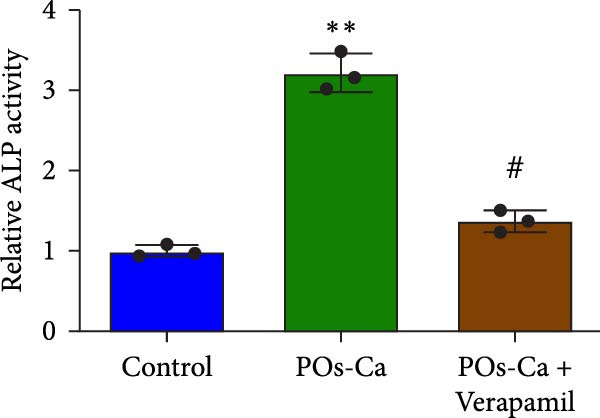
(B)

**Figure:**
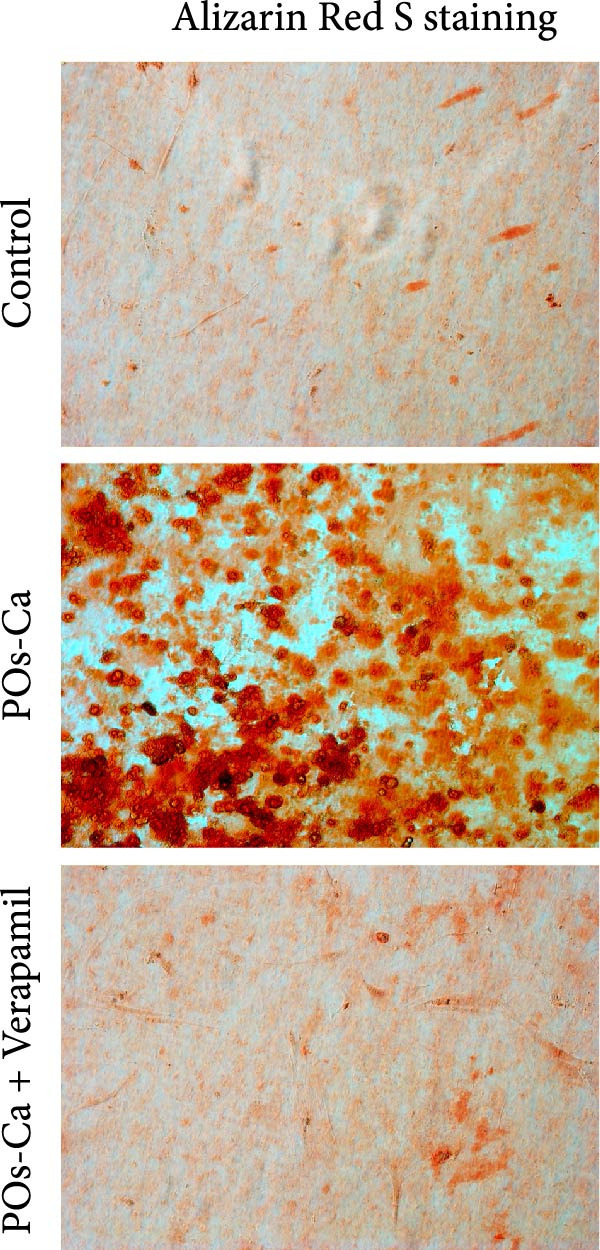
(C)

**Figure:**
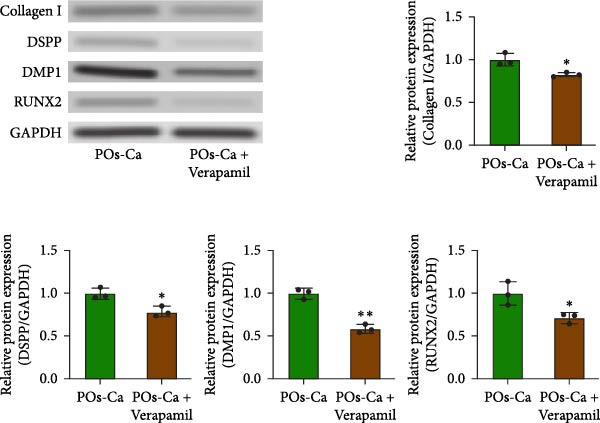
(D)

Functionally, POs‐Ca treatment significantly enhanced early osteogenic activity in hDPSCs, as evidenced by a 3.22‐fold increase in ALP activity on day 7 compared to the control group (*p* < 0.01, Figure 3B). Verapamil pretreatment markedly suppressed this POs‐Ca‐induced ALP activity elevation (*p* < 0.05), indicating calcium influx as essential for this early osteogenic response. Similarly, Alizarin Red S staining performed on day 21 showed that POs‐Ca significantly promoted the formation of mineralized nodules, an indicator of late‐stage osteogenic maturation. This enhanced mineralization was significantly reduced by verapamil cotreatment (Figure 3C). Western blot analysis further corroborated these findings, showing that verapamil attenuated the upregulation of key osteogenic proteins induced by POs‐Ca, including Collagen I, DSPP, DMP1, and RUNX2 (Figure 3D). Collectively, these results indicated that POs‐Ca markedly promotes both the initiation and maturation phases of osteogenic differentiation in hDPSCs. Critically, calcium influx acts as a key mediator in initiating the osteogenic effects of POs‐Ca.

### 3.4. AMPK Activation Is Essential for Osteogenic Enhancement in hDPSCs

Given the role of calcium signaling in energy sensing pathways, we next explored whether AMPK, a calcium‐responsive metabolic sensor, is involved in POs‐Ca‐mediated effects. Western blot analysis revealed that POs‐Ca treatment robustly activated the AMPK pathway, as evidenced by significantly increased the phosphorylation levels of AMPK (p‐AMPK) and its downstream target ULK1 (p‐ULK1). However, cotreatment with CC, a pharmacological AMPK inhibitor, markedly suppressed POs‐Ca‐induced activation of p‐AMPK and p‐ULK1 (Figure 4A), confirming AMPK pathway activation by POs‐Ca.

Figure 4AMPK activation mediates POs‐Ca‐induced osteogenic differentiation and autophagy in hDPSCs. (A) Western blot analysis of AMPK signaling (p‐AMPK), autophagy markers (p‐ULK1 and LC3), and osteogenic markers (Collagen I, DSPP, DMP1, and RUNX2) in control, POs‐Ca‐treated (5 mg/mL), and POs‐Ca + CC‐treated hDPSCs after 7 days of osteogenic induction. (B) ALP activity in control, POs‐Ca‐treated, and POs‐Ca + CC‐treated hDPSCs after 7 days. (C) Representative Alizarin Red S staining of mineralized matrix deposition in control, POs‐Ca‐treated, and POs‐Ca + CC‐treated hDPSCs after 21 days. Scale bar = 100 μm. (D) Representative LC3‐GFP fluorescence images showing autophagosome formation (puncta) in control, POs‐Ca‐treated, and POs‐Ca + CC‐treated hDPSCs after 24 h. Scale bar = 20 μm. (E) Representative transmission electron microscopy (TEM) images of autophagosome formation in control, POs‐Ca‐treated, and POs‐Ca + Compound C (CC)‐treated hDPSCs after 48 h (scale bar = 500 nm). Red arrows indicate autophagosomes. (F) Quantification of autophagosomes per cell in panel E. Data were presented as mean ± SD, with experiments repeated three times.  ^∗^ *p* < 0.05 compared to control;  ^∗∗^ *p* < 0.01 compared to control; #*p* < 0.05 compared to POs‐Ca group; ##*p* < 0.01 compared to POs‐Ca group.

**Figure:**
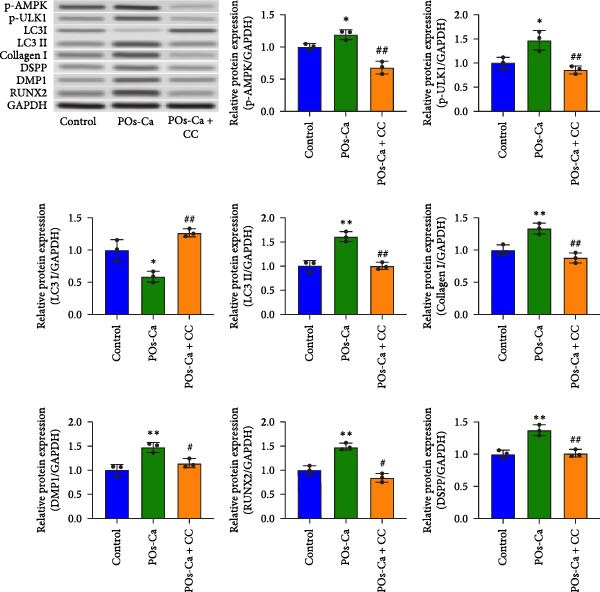
(A)

**Figure:**
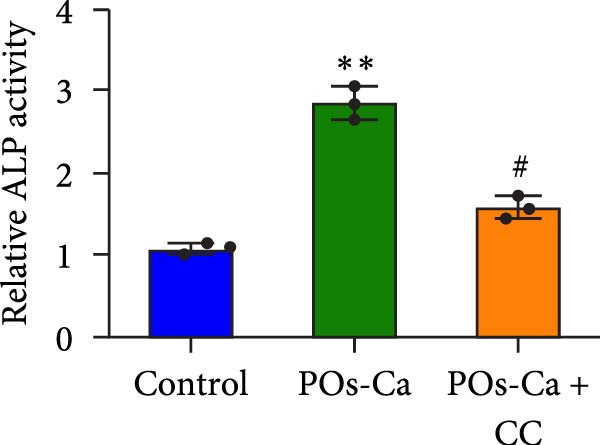
(B)

**Figure:**
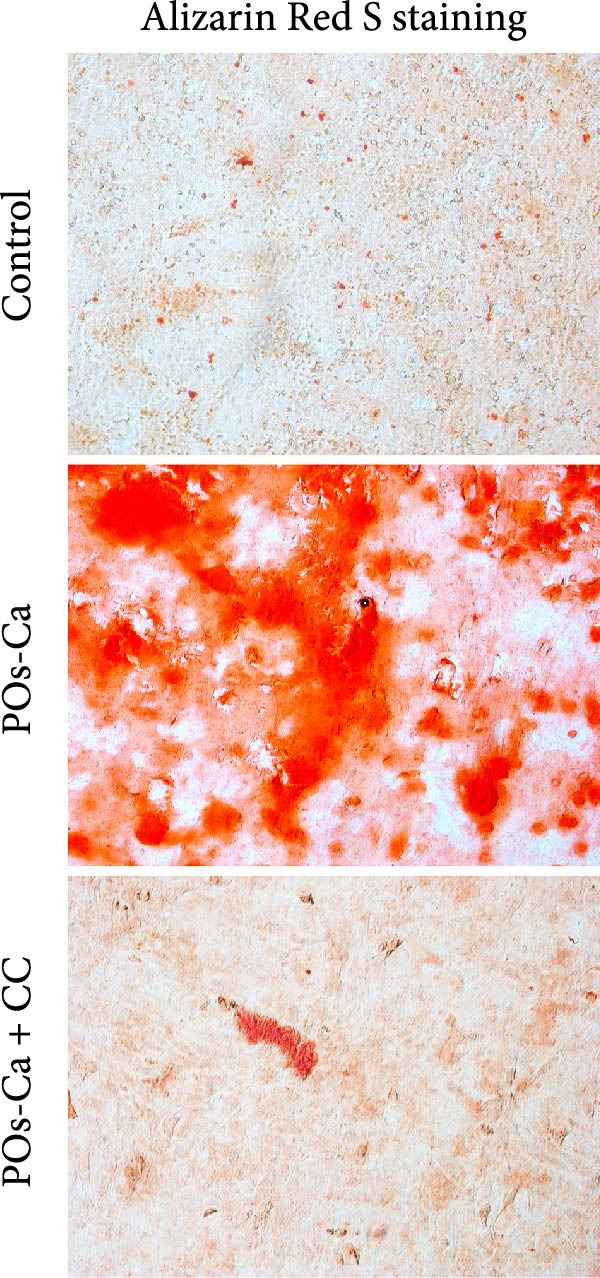
(C)

**Figure:**
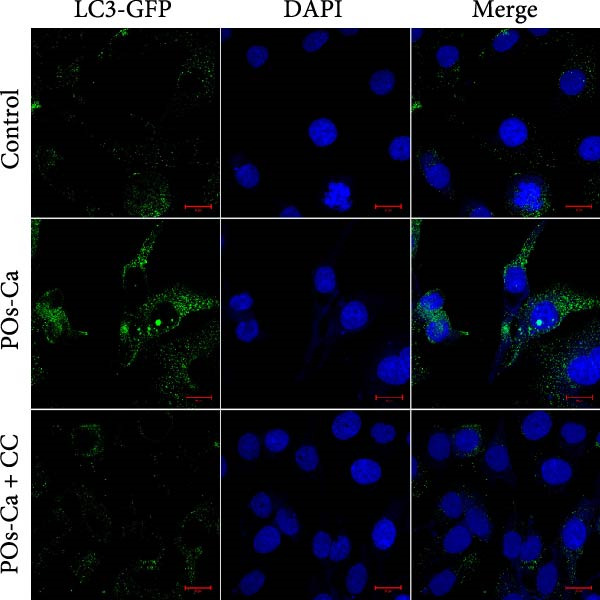
(D)

**Figure:**
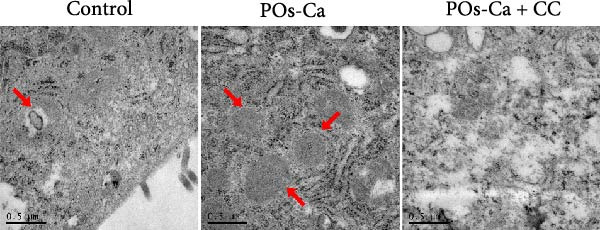
(E)

**Figure:**
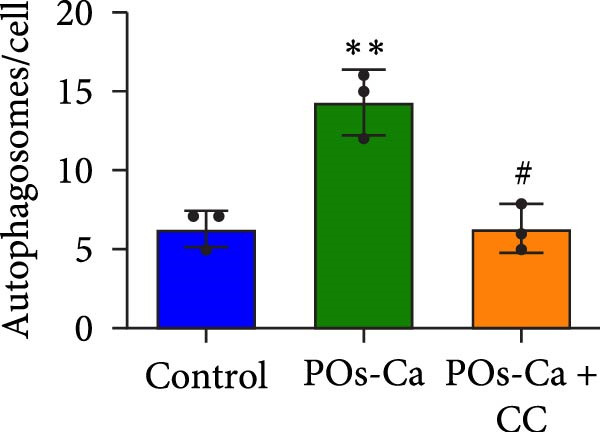
(F)

To evaluate the functional significance of AMPK activation in POs‐Ca‐mediated osteogenesis, we assessed key differentiation markers under AMPK inhibition. After 7 days of induction, POs‐Ca treatment resulted in a significant elevation of ALP activity, which was significantly blunted by CC co‐treatment (Figure 4B). Similarly, Alizarin Red S staining on day 21 demonstrated extensive mineral deposition in the POs‐Ca group, whereas CC cotreatment markedly suppressed this mineralization (Figure 4C). Consistent with these functional assays, western blot analysis confirmed that POs‐Ca markedly upregulated the expression of key osteogenic markers (Collagen I, DSPP, DMP1, and RUNX2), while cotreatment with CC substantially diminished their expressions (Figure 4A). Together, these results demonstrate that AMPK activation is indispensable for POs‐Ca‐induced osteogenic differentiation in hDPSCs.

### 3.5. AMPK‐Dependent Autophagy Drives POs‐Ca‐Induced Osteogenic Differentiation in hDPSCs

Intriguingly, POs‐Ca treatment enhanced the expression of the autophagy marker LC3‐II compared to controls. Furthermore, AMPK inhibition via CC also affected autophagy marker levels (Figure 4A), suggesting a link. These findings were further supported by LC3‐GFP fluorescence microscopy imaging, which revealed abundant LC3 punctate structures in the cytoplasm of POs‐Ca‐treated cells, indicative of enhanced autophagic flux. This effect was markedly attenuated upon CC treatment (Figure 4D). Similarly, TEM showed a pronounced increase in autophagosome formation in POs‐Ca‐treated cells, while CC cotreatment reduced the number of autophagosomes to levels comparable with the control group (Figure 4E,F).

**Figure 5:**
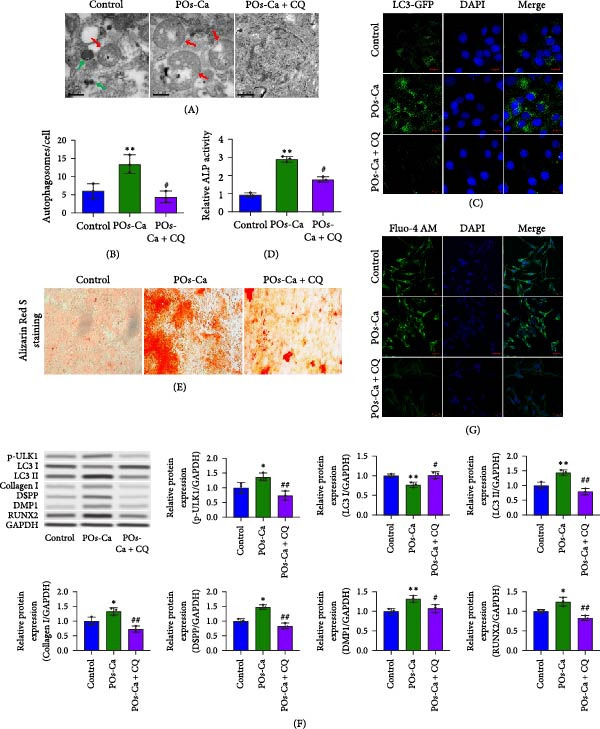
AMPK‐dependent autophagy mediates POs‐Ca‐induced osteogenesis and exhibits feedback regulation of calcium signaling in hDPSCs. (A) Representative transmission electron microscopy (TEM) images of autophagosome formation in control, POs‐Ca‐treated (5 mg/mL), and POs‐Ca + CQ‐treated hDPSCs after 48 h (scale bar = 500 nm). Red arrows indicate autophagosomes and green arrows show lysosomes. (B) Quantification of autophagosomes per cell from panel A confirms that POs‐Ca promotes autophagy, while CQ significantly inhibits autophagosome accumulation. (C) Representative LC3‐GFP fluorescence images showing autophagosome formation (puncta) in control, POs‐Ca‐treated, and POs‐Ca + CQ‐treated hDPSCs after 24 h. (D) ALP activity in control, POs‐Ca‐treated, and POs‐Ca + CQ‐treated hDPSCs after 7 days. (E) Representative Alizarin Red S staining of mineralized matrix deposition in control, POs‐Ca‐treated, and POs‐Ca + CQ‐treated hDPSCs after 21 days (scale bar = 100 μm). (F) Western blot analysis of autophagy markers (p‐ULK1 and LC3) and osteogenic‐related proteins (Collagen I, DSPP, DMP1, and RUNX2) in control, POs‐Ca‐treated, and POs‐Ca + CQ‐treated hDPSCs after 7 days. (G) Representative Fluo‐4 AM fluorescence images showing intracellular calcium levels in control, POs‐Ca‐treated, and POs‐Ca + CQ‐treated hDPSCs after 24 h. Data were presented as mean ± SD, from three independent experiments.  ^∗^ *p* < 0.05 compared to control;  ^∗∗^ *p* < 0.01 compared to control; #*p* < 0.05 compared to POs‐Ca group; ##*p* < 0.01 compared to POs‐Ca group.

Given the established role of autophagy as a crucial downstream effector of AMPK signaling [36], and its essential function in stem cell osteogenesis [37–40], we next blocked autophagic flux with CQ(5 µM). Ultrastructural analysis by TEM confirmed CQ attenuated POs‐Ca‐induced autophagosome formation (Figure 5A,B). Consistently, LC3‐GFP observation showed that puncta formation was diminished by CQ (Figure 5C). Western blotting confirmed that CQ potently suppressed ULK1 phosphorylation and LC3‐II level induced by POs‐Ca treatment (Figure 5F).

To assess the functional relevance of POs‐Ca‐induced autophagy during osteogenic differentiation, we employed CQ to pharmacologically inhibit autophagic flux. After 7 days of treatment, CQ significantly attenuated the POs‐Ca‐induced enhancement of ALP activity, reducing it to 1.87‐fold of baseline (*p* < 0.05 versus POs‐Ca alone, Figure 5D). Consistently, Alizarin Red S staining at day 21 revealed that CQ markedly suppressed POs‐Ca‐enhanced matrix mineralization (Figure 5E). Moreover, western blot analysis at day 7 confirmed that CQ cotreatment substantially reduced the upregulation of key osteogenic markers, including Collagen I, DSPP, DMP1, and RUNX2, induced by POs‐Ca (Figure 5F). Strikingly, Fluo‐4 AM imaging revealed CQ reduced POs‐Ca‐induced intracellular Ca^2+^ influx (Figure 5G). This unexpected result suggests a novel bidirectional regulatory relationship between autophagy and Ca^2+^ signaling.

Together, these findings indicate that POs‐Ca promotes the osteogenic differentiation of hDPSCs, at least in part, through the AMPK‐ULK1‐autophagy axis, with autophagy acting as a critical downstream mediator. Moreover, the observed suppression of Ca^2+^ influx by autophagy inhibition highlights a previously unrecognized feedback mechanism, in which autophagy may sustain or amplify calcium signaling during osteoinduction.

## 4. Discussion

hDPSCs represent a promising frontier in regenerative dentistry, yet their clinical translation remains hampered by the limitations of current osteogenic strategies reliant on supraphysiological growth factors. To address this critical gap, our study introduces POs‐Ca as a novel bioactive agent that leverages physiological calcium signaling to promote osteogenic differentiation of hDPSCs. Unlike traditional calcium phosphates such as TCP or polyphasic calcium phosphates (Poly‐CaP), which act mainly as passive scaffolds and dissolve slowly [41, 42], POs‐Ca is highly water‐soluble and rapidly releases bioavailable Ca^2+^. Importantly, POs‐Ca is a biologically derived from food‐grade oligosaccharides and calcium salts, offering distinct advantages in terms of safety and cost‐effectiveness compared to synthetic alternatives. This material facilitates robust intracellular Ca^2+^ influx at biocompatible concentrations (5 mg/mL, below IC_50_ of 6.68 mg/mL), without compromising viability or inducing apoptosis in hDPSCs, directly engaging calcium’s role as a second messenger in osteoblastic differentiation [43].

A key finding of our study is the elucidation of a hierarchical signaling axis where POs‐Ca‐induced Ca^2+^ influx serves as the molecular initiator. We demonstrate that this Ca^2+^ entry sensitive to verapamil activates AMPK phosphorylation via CaMKK *β* [44], positioning AMPK as a critical signal integrator that translates ionic stimuli into metabolic commands. Activated AMPK subsequently phosphorylates ULK1, triggering pro‐osteogenic autophagic flux, as evidenced by increased LC3‐II conversion, autophagosome formation (Figure 4A,D,E), and the accumulation of cytoplasmic puncta. These autophagic processes are essential not only for organelle quality control but also for the generation of biosynthetic precursors during differentiation. Crucially, we uncovered a paradoxical yet novel regulatory feature: inhibition of autophagy by CQ not only suppressed osteogenic marker expression but also significantly reduced POs‐Ca‐induced Ca^2+^ influx (Figure 5G). We speculate that this bidirectional crosstalk may be mediated by autophagy‐dependent maintenance of calcium‐storing organelles such as the endoplasmic reticulum and mitochondria. This paradigm‐shifting observation extends beyond autophagy’s known role in mitigating endoplasmic reticulum stress [36, 45] by establishing its active participation in ionic homeostasis, forming a self‐amplifying loop that potentiates osteogenesis. Such findings suggest that POs‐Ca may simultaneously function as a calcium donor and an intracellular modulator, offering dual‐mode regulation that enhances both signal initiation and cellular adaptation.

The pivotal role of AMPK in this axis warrants particular emphasis. Classically, AMPK functions as an energy sensor, regulating metabolic balance by inhibiting anabolic pathways and promoting catabolic processes. In MSCs, AMPK activation favors osteogenesis partly by inhibiting mTOR signaling, stimulating autophagy, enhancing mitochondrial function, and suppressing adipogenic differentiation [36, 46, 47]. Our data reinforce and extend this understanding by demonstrating that AMPK is a nonredundant mediator of calcium‐induced osteogenic differentiation, acting as a mechanistic bridge between extracellular Ca^2+^ signals and intracellular degradation machinery. Inhibition of AMPK signaling by CC severed this link, abolishing both autophagy and osteogenesis (Figure 4), confirming AMPK’s irreplaceable function. This expands the biological significance of AMPK beyond energy sensing, positioning it as a metabolic–ionic interface that coordinates differentiation through autophagy‐dependent resource allocation.

POs‐Ca’s mechanism of action reveals compelling translational distinctions from existing osteoinductive agents. Unlike BMP‐2—which risks ectopic ossification and imposes high costs [48, 49]—POs‐Ca exploits endogenous calcium‐sensing machinery for targeted activation. Contrasting lithium’s neurotoxic liabilities [50], it engages AMPK‐mediated metabolic precision. Where *β*‐glycerophosphate acts as a passive phosphate donor, POs‐Ca delivers synergistic Ca^2+^/oligosaccharide bioactivity. Furthermore, its simple, low‐cost production process and favorable safety record (originating from food‐grade compounds) make POs‐Ca particularly suited for scalable manufacturing and clinical application. These features collectively position POs‐Ca as a safe, economically viable, and biologically effective alternative for bone and dental tissue regeneration.

Clinically, our findings open new avenues for utilizing POs‐Ca in minimally invasive regenerative therapies. In dentistry, POs‐Ca could be formulated into injectable gels or hydrogels for direct pulp capping, pulp revitalization, or apical barrier induction in immature permanent teeth. Its ability to stimulate hDPSCs differentiation without exogenous growth factors presents a cost‐effective solution for pulp regeneration. Beyond endodontics, POs‐Ca holds promise for alveolar bone preservation post‐extraction, periodontal defect repair, and implant site preparation. In orthopedic contexts, its application could be extended to craniofacial defect filling, guided bone regeneration, or as an adjuvant in bone graft materials. Future studies incorporating in vivo efficacy and biomaterial integration will be critical for clinical translation.

While our findings illuminate a previously unrecognized signaling paradigm, certain limitations frame future research priorities. The focus on hDPSCs, though clinically relevant for dental regeneration, invites exploration of this axis in other MSC populations (e.g., adipose‐derived). The specific calcium channels (e.g., CaSR and TRPV4) warrant genetic validation beyond pharmacological inhibition. Unresolved questions persist regarding how AMPK‐autophagy coordinates metabolic reprogramming during differentiation—a process potentially measurable through glycolytic/OXPHOS flux analysis. Ultimately, bridging these in vitro insights with in vivo models will be essential to confirm the translational feasibility of POs‐Ca–based therapies.

## 5. Conclusions

In conclusion, this work delineates a fundamentally new regulatory circuit in which POs‐Ca drives hDPSCs osteogenesis through a calcium‐initiated AMPK‐autophagy cascade. The discovery of reciprocal Ca^2+^‐autophagy crosstalk reshapes our understanding of how organelle homeostasis dynamically integrates with ionic signaling to control stem cell commitment. Beyond advancing mechanistic knowledge, these insights establish a rational design blueprint for next‐generation biomaterials that exploit calcium‐autophagy synergism—such as POs‐Ca/rapamycin hydrogels for minimally invasive pulp regeneration—while offering metabolic sensors as novel therapeutic targets in bone and dental engineering.

## Disclosure

All authors have read and agreed to the published version of the manuscript.

## Conflicts of Interest

The authors declare no conflicts of interest.

## Author Contributions

Jiayuan Zhang conceived and designed research. Jiayuan Zhang and Yunqing Liu performed experiments. Shuhei Hoshika and Chiharu Kawamoto analyzed data. Hidehiko Sano interpreted results of experiments. Jiayuan Zhang prepared first draft of the manuscript. Atsushi Tomokiyo and Jie Gao approved latest version of the manuscript.

## Funding

This work was financially supported by the Grants‐in‐Aid for Scientific Research from the Japan Society for the Promotion of Science (Grants JP25K13014, JP25K22677 and JP22K09994), awarded to Atsushi Tomokiyo and Hidehiko Sano, respectively. This work was also supported by the JST SPRING program (Grant JPMJSP2119), which was awarded to Jiayuan Zhang.

## Data Availability

The data supporting the findings of this study are available within the article. Additional data may be provided upon reasonable request to the corresponding author.
